# Molecular mechanisms of long noncoding RNAs associated with cervical cancer radiosensitivity

**DOI:** 10.3389/fgene.2022.1093549

**Published:** 2023-01-04

**Authors:** Shuying Wu, He Zhu, Yishi Wu, Cong Wang, Xuefeng Duan, Tianmin Xu

**Affiliations:** Department of Obstetrics and Gynecology, Second Hospital of Jilin University, Changchun, China

**Keywords:** cervical cancer, long noncoding RNA (lncRNA), radiosensitive, DNA damage repair, apoptosis

## Abstract

Despite advances in cervical cancer screening and human papilloma virus (HPV) vaccines, cervical cancer remains a global health burden. The standard treatment of cervical cancer includes surgery, radiation therapy, and chemotherapy. Radiotherapy (RT) is the primary treatment for advanced-stage disease. However, due to radioresistance, most patients in the advanced stage have an adverse outcome. Recent studies have shown that long noncoding RNAs (lncRNAs) participate in the regulation of cancer radiosensitivity by regulating DNA damage repair, apoptosis, cancer stem cells (CSCs), and epithelial–mesenchymal transition (EMT). In this review, we summarize the molecular mechanisms of long noncoding RNAs in cervical cancer and radiosensitivity, hoping to provide a theoretical basis and a new molecular target for the cervical cancer RT in the clinic.

## Introduction

Cervical cancer is the fourth most common female lower genital tract malignancy, and over 570000 women are diagnosed with cervical cancer each year (Ferlay et al., 2019). The staging system of the International Federation of Gynecology and Obstetrics (FIGO) determines the clinical decision of cervical cancer. Patients with cervical cancer in its early stages (stages I, II) usually undergo surgical treatment, and those who have risk factors will receive adjuvant radiotherapy (RT). Patients with advanced cervical cancer (stage III, IV) or patients with severe complications or surgical contraindications will receive RT as the main treatment (Meng et al., 2018). Approximately three-fifths of all cervical cancer patients undergo RT (Chung et al., 2005). RT has greatly improved therapeutic effectiveness in cervical cancer. However, in the clinic, some patients experience radioresistance, and those patients often suffer an unfavorable treatment outcome. Hence, enhancing the radiosensitivity of cervical cancer cells is important for patients. Researchers have looked for various strategies to solve this urgent problem. In recent years, researchers have found that long noncoding RNAs (lncRNAs) have a crucial function in regulating cervical cancer radioresistance.

LncRNAs are a class of noncoding RNAs that are longer than 200 nucleotides and have a complex function in regulating biological processes, including cell proliferation, migration, invasion, the cell cycle and apoptosis (Esteller, 2011). Recent studies suggest that lncRNAs interact with DNA, RNA, and protein, thus participating in a variety of cellular processes, including transcription, translation, and posttranscriptional regulation (Huarte et al., 2010). LncRNAs can affect gene expression epigenetically *via* diverse mechanisms: 1) lncRNAs can regulate histone modification and chromatin status to affect gene transcription. 2) lncRNAs can attract transcription factors or repressors to the promoter of a particular gene (Long et al., 2017), 3) lncRNAs can prevent transcription-related proteins from binding to their DNA targets by acting as decoys. LncRNAs can compete with endogenous RNAs to silence target genes by binding to miRNAs and blocking their function (Gutschner and Diederichs, 2012). Many studies have demonstrated that there is an association between lncRNAs and radiosensitivity of malignant tumors (Yang Q. et al., 2019; Tang T. et al., 2019; Yang Z. et al., 2019; Liu R. et al., 2020; Wang et al., 2020). However, there is no summary of the systematic and comprehensive molecular mechanisms of long noncoding RNAs associated with cervical cancer radiosensitivity. Hence, we performed a systematic literature review on lncRNAs and the detailed mechanisms by which they enhance or weaken the radiosensitivity of cervical cancer, and we discuss these topics in seven sections.

Radiation causes DNA damage, inhibits cell proliferation, and induces cellular apoptosis to treat cancer (Yao et al., 2019). The conventional radiobiological principles proposed by Withers are known as the “4Rs”, repair of sublethal cellular damage, reoxygenation of cells within the cell cycle, redistribution of the surviving cells and repopulation of cells after radiation (Withers, 1975). Radiotherapy induces tumour cell death by causing cell cycle arrest and leading to DNA damage. Repair of sublethal cellular damage and repopulation of cells after radiation make tumor cells resistant to radiation and decrease radiosensitivity; on the other hand, reoxygenation of cells within the cell cycle and redistribution of the surviving cells decrease the radiosensitivity of tumor cells (Brown et al., 2014).

**FIGURE 1:**
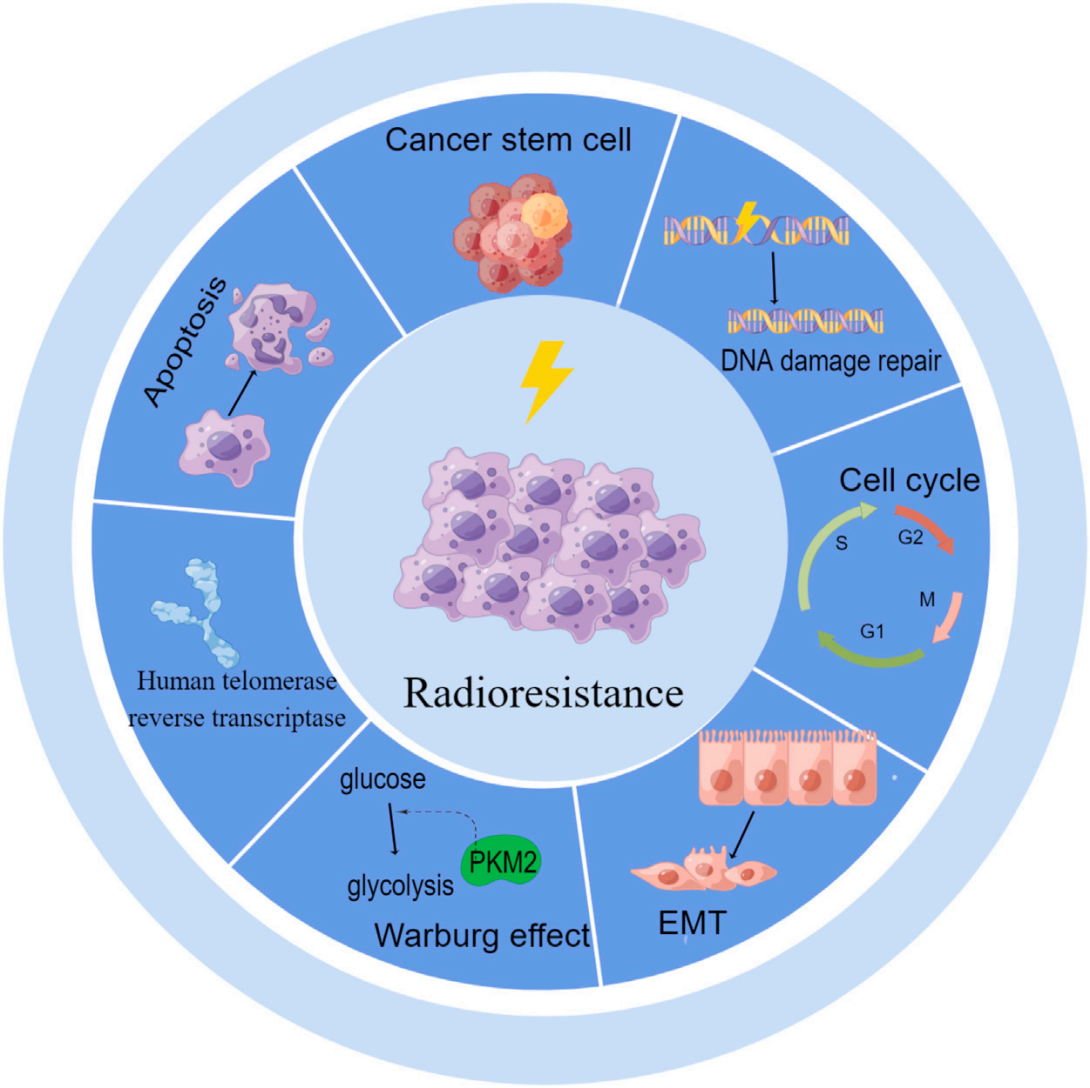
LncRNAs regulate radioresistance by DNA damage repair, cell cycle, apoptosis, EMT, CSCs, aerobic glycolysis and human telomerase reverse transcriptase.

Numerous studies have shown that lncRNAs are functionally involved in various critical cellular processes that regulate RT in cervical cancer. LncRNAs can modulate DNA damage repair, the cell cycle, and apoptosis and can also influence the redistribution of surviving cells by regulating EMT, cancer stem cells (CSCs) and the reoxygenation of cells by regulating aerobic glycolysis after RT in cervical cancer (Figure 1).

## LncRNAs modulate DNA damage repair and the cell cycle

Radiation-induced DNA damage includes base damage, single- and double-strand breaks, and DNA crosslinks caused by direct ionization or free oxygen radical effects (Olive, 1998), DNA double-strand breaks (DSBs) are the most critical. When DNA damage occurs, the cell cycle checkpoint is active, causing a delay in the cell cycle, and providing time to repair DNA damage. Finally, damage that cannot be repaired will induce cancer cell apoptosis. Different DNA repair pathways initiate and repair different types of DNA damage. However, there is also functional overlap between different DNA repair pathways (Mao and Wyrick, 2016). Among these repairs are nonhomologous end joining (NHEJ) repair, homologous recombination (HR) nucleotide excision repair, mismatch repair, and base excision repair (BER) (Sancar, 1995; Hoeijmakers, 2001; Schärer, 2003; Chapman et al., 2012; Krokan and Bjørås, 2013; Mao and Wyrick, 2016). In mammalian cells, ataxia telangiectasia-mutated (ATM) and ATM and Rad-3 related (ATR) are two phosphatidylinositol kinases that are involved in these pathways (Bakkenist and Kastan, 2004). DNA damage and DNA repair pathways are crucial determinants of radiosensitivity.

In mammalian cells, ATM and ATR are two phosphatidylinositol kinases that are involved in DNA damage repair (Bakkenist and Kastan, 2004). When DNA damage occurs, the MRE11-RAD50-NBS1 (MRN) complex first detects damaged DNA and recruits ATM to damage sites (Lee and Paull, 2004). Then ATM is activated by autophosphorylation at Ser 1981 (Bakkenist and Kastan, 2003) and acetylation at K3106 (Sun et al., 2005). Activated ATM can also activate p53-dependent and independent pathways to initiate the downstream protein kinase phosphorylation cascade (Shiloh and Ziv, 2013). In p53-independent pathways, activated ATM phosphorylates downstream targets Chk2 at Thr68 and leading to phosphorylation of Cdc25 at Ser 216. Phosphorylated Cdc25 bind to 14-3-3 proteins and sequestrate Cdc25 in cytoplasm inhibiting activation of cyclin B/Cdk1 by Cdc25 and resulting in G2/M arrest. In p53 dependent pathways, the p53 protein activates downstream genes such as p21 which can inhibit CDK2 resulting in G1 arrest, and 14-3-3, which can inhibit cyclin B/Cdk1, resulting in G2/M arrest. Teng et al. (Teng et al., 2015) revealed that after IR-induced DNA damage, ATM activation activates ATR, and then Chk1 is phosphorylated at Ser345. ATR-Chk1 restricts CDK2 activity and finally cause G1 arrest. Through the above steps, Cell cycle arrest provide more time for DNA damage repair. This will cause radioresistance. Therefore, the use of checkpoint inhibitors to improve the therapeutic effects of radiotherapy is a clinical treatment strategy in the future.

ATM has been proved to be connected with therapeutic effects of radiotherapy in cervical cancer. Roossink et al. (2012) recruited 349 advanced stage cervical cancer patients to detect the expression of p-ATM in their pretreatment tissue using immunohistochemical analysis and results revealed that 344 patients (98.6%) had positive nuclear staining and 183 patients (52.4%) had high expression. By analyzing clinical data, they found that high p-ATM expression was related to poor survival. To determine the relationship between p-ATM and the response of cervical cancer to irradiation, they carried out *in vitro* studies and found that the expression of activated ATM was high in Caski cells even before irradiation while in HeLa and SiHa cells was extremely low. And in comparison to HeLa and SiHa cells, Caski cells were significantly more resistant to irradiation. This result reveals us there is a positive correlation between baseline level of p-ATM and radioresistance. Then they proved ATM inhibition led to the destroy of G2/M arrest and finally sensitized tumour cells to radiation. The same result was also reported by. Teng et al. (2015) They use inhibitors of ATR (ETP-46464) (Hickson et al., 2004; Toledo et al., 2011) and ATM (KU55933) to treat Hela and SiHa cervical cancer cells for 15 min before IR exposure and then assessed clonogenic survival. Both ETP-46464 and KU55933 could enhance the response to IR by affecting the expression levels of p-ATM/P-Chk1 and p-ATM/P-Chk2. A compound named Ro 90-7501 can enhance the adiosensitivity of cervical cancer by inhibiting ATM (Tamari et al., 2019), A Chinese herbal medicine Osthole can also inhibited phosphorylation of ATM and enhances irradiation sensitivity of cervical cancer cells (Che et al., 2018).

**FIGURE 2:**
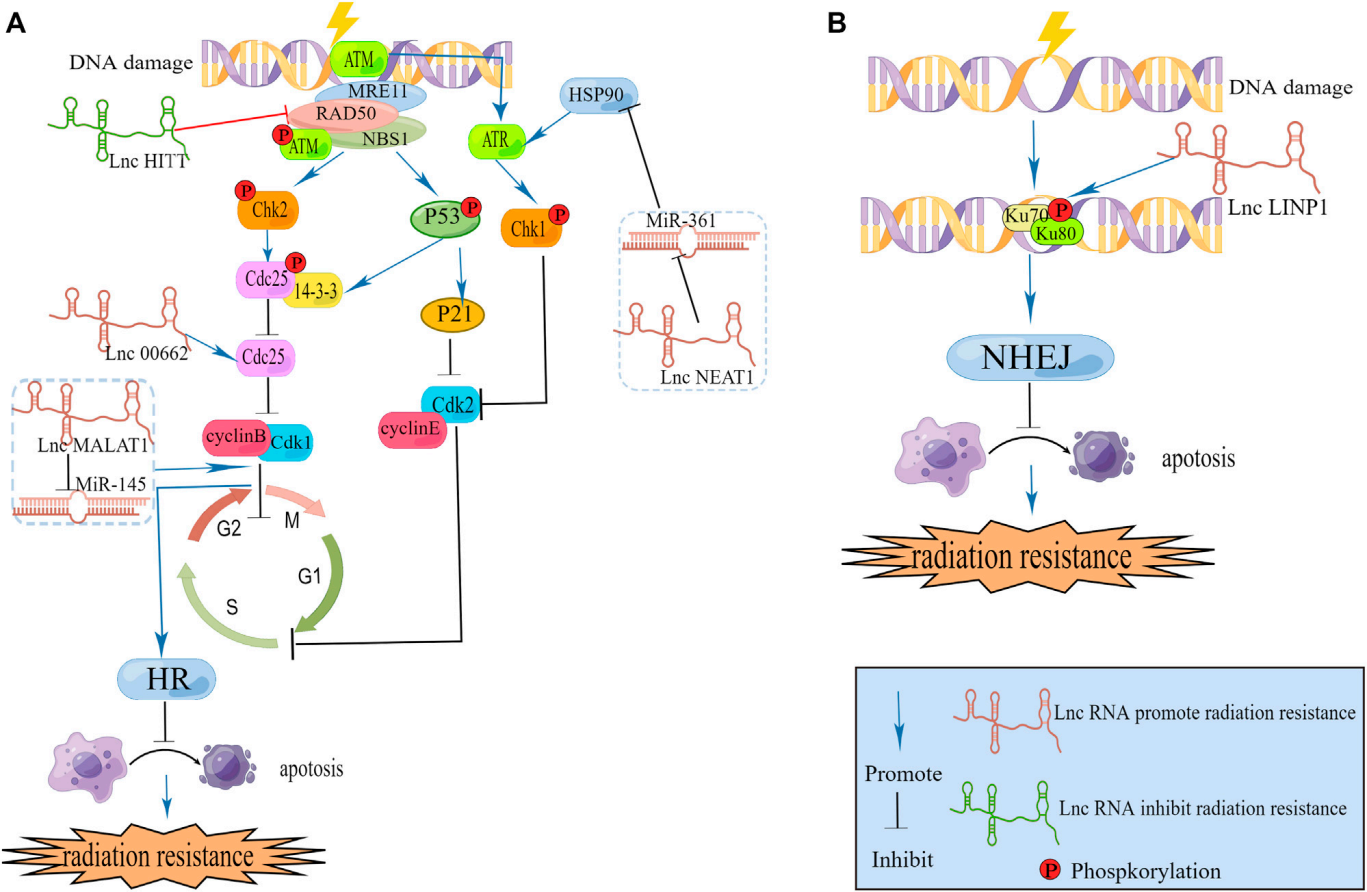
**(A) **LncRNAs modulate DNA damage repair caused by HR and cell cycle. **(B)** LncRNA modulate DNA damage repair caused by NHEJ.

Many studies showing that lncRNAs can directly act on DNA damage repair-related protein to regulate DNA damage response. We retrieved related literature and found that lncRNAs can regulate the expression of ATM in various tumors, including cervical cancer (Sharma et al., 2020), esophageal squamous cell cancer (Chen et al., 2018; Zhang et al., 2019), pancreatic cancer (Chen et al., 2017), and thyroid cancer (Yuan et al., 2020). Zhao et al. (2020) identified an lncRNA named HITT that maps to 14q32, and contains three exons. LncRNA HITT inhibits ATM activity by affecting MRN. ATM inhibitors may serve as an attractive target to overcome radioresistance in cervical cancer (Figure 2).

Kyungsoo Ha c (Ha et al., 2011) found in cervical cancer that ATR, which is a *bona fide* heat shock protein (hsp) 90 client protein, is downregulated when hsp90 is inhibited. Additionally, they found that treatment with hsp90 inhibitors decreased both ATR and CHK1 levels, and the hsp90 inhibitor increased radiation-induced DNA damage as well as apoptosis of transformed cells by blocking ATR-CHK1-mediated DNA damage repair signaling. Xu et al. (2020) carried out a luciferase reporter assay and demonstrated that in cervical cancer, miR-361 reduces the expression of hsp90 directly, and lncRNA NEAT1. Directly downregulates the expression of miR-361. From the two above studies, we can establish a link between the lncRNAs NEAT1, hsp90, ATR and radiation-mediated DNA damage repair. We infer that lncRNA NEAT1 could increase the expression of hsp90 and ATR, DNA damage repair, causing radioresistance. Han et al. (2018) have already revealed that NEAT1 can facilitate radioresistance in cervical cancer. They use the luciferase reporter analysis and RNA immunoprecipitation (RIP) assay to prove lncRNA NEAT1 can spong miR-193b-3p as a ceRNA to regulate cyclin D1. More experiments are needed to investigate this hypothesis.

As we mentioned above CDC25 is required for DNA damage repair. LINC00662 is reported to be connected with CDC25A in cervical cancer. Wei et al. (2020) collected 39 samples from patients (cancer samples and adjacent cervical samples) with cervical cancer and found that the expression of LINC00662 was markedly elevated in cervical cancer tissues in comparison with adjacent tissues. They subsequently used two CC cell lines (C33A and Caski) to investigate the function of LINC00662. According to their findings, LINC00662 facilitated cervical cancer progression and radioresistance *via* miR-497-5p adsorption and upregulation of CDC25A. However they did not compare LINC00662 expression between different patients’ tumor tissues and collected clinical information to assess the therapeutic effects of radiotherapy in different expression level. Follow-up researchers can supplement the above points if they carry out experiments in this area, which can significantly improve the quality of the article.

LncRNA in nonhomologous end joining pathway 1 (LINP1) is located on chromosome 10p14 and is associated with the NHEJ repair pathway (Thapar et al., 2020). The NHEJ repair pathway is a major DNA damage repair pathway involved in the repair of DSBs in DNA that are damaged after radiotherapy (Lord and Ashworth, 2012). Wu et al. (2020) collected twenty paired cervical cancer tissues and adjacent normal tissues from Guizhou Provincial People’s Hospital (Guizhou, China) to examine each tissue’s LINP1 expression level and revealed that CC tissues exhibit higher expression of LINP1 than adjacent noncarcinoma tissues and that high expression of LINP1 can suppress KLF2 and PRSS8 to aggravatecervical cancer development. Similarly, Wang et al. (2018) isolated total RNA from five tissues of multiple patients and then carried out qRT‒PCR experiments. In comparison to adjacent tissues, tumor tissues from cervical cancer patients contained higher levels of LINP1. They found that within 30 min of RT, LINP1 translocates into the nucleus, and the protein was upregulated later. We know that RT can cause DNA DSBs and then Ku70–Ku80 heterodimers are recruited and activated by NHEJ. Additionally, by promoting NHEJ protein recruitment to DSBs, Ku80 can bind breaks in DNA. One of the Ku-interacting proteins called DNA-PKCs is a serine/threonine-protein kinase required for NHEJ in human cells; Wang et al. (2018) revealed that LINP1 associates with Ku80 and DNA-PKCs in cervical cancer cell lines. Moreover, LINP1-deficient cells were more prone to radiation-induced cell death and DNA DSBs after RT than control cells. Together, these data indicated that in cervical cancer, the lncRNA LINP1 contributes to radiation resistance by increasing the efficiency of DNA damage repair through the NHEJ pathway (Figure 2).

DNA damage induced by radiation induces DNA repair pathways and alters the expression of cell cycle checkpoint molecules. Affected cells are able to arrest cell cycle progression and repair damaged DNA or undergo apoptosis if the damage cannot be repaired. Radiosensitivity is closely associated with cell cycle arrest, since different phases of the cell cycle exhibit different levels of radiosensitivity, and the G2/M phase is the most radiosensitive phase of the cell cycle (Wilson, 2004). LncRNAs regulate radiosensitivity in cervical cancer by arresting cell cycle growth to interfere with DNA damage repair. G0/G1 phase Han et al. (2018) found that in cervical cancer, the absence of NEAT1 leads to cell cycle arrest in G0/G1, and this change allows cells to undergo apoptosis. This is because NEAT1 binds to miR-193b in a competitive manner to regulate the expression of CCND1, enhancing radioresistance in cervical cancer. In another study, Jiang et al. (2014) investigated lncRNA MALAT1 in cervical cancer and found that MALAT1 downregulation causes cells to enter the G1 phase and that cyclin D1, cyclin E, and CDK6 levels are significantly altered. Other researchers Lu et al. (2016) reported that lncRNA MALAT1 can regulate the cell cycle to influence radiosensitivity. They revealed that lncRNA MALAT1 was negatively correlated with miR-145, which can enhance G2/M phase block. These two studies provide evidence that the function of MALAT1 in cervical cancer is related to the cell cycle. In the future, we can investigate more lncRNAs that are connected with the cell cycle to learn their roles in regulating radiosensitivity in cervical cancer.

## LncRNAs modulate apoptosis

Among the mechanisms of irradiation-induced toxicity, apoptosis plays a significant role. Tumor cells that undergo apoptosis display a number of morphological features, including condensed chromatin, shrinkage and fragmentation, blebbing of the plasma membrane, and the formation of apoptotic bodies (Wyllie, 2010). Extrinsic death receptor signals and intrinsic mitochondrial signals are the two main inducers of apoptosis.

To determine the changes in signaling pathways in cervical cancer cells after radiation, (Khalilia et al., 2018) exposed cervical cancer cells to different doses of radiation and revealed that apoptosis, RAS, TGF-β, WNT, the oxidative stress response, and p53 were significant downstream signals. Among these pathways, the p53 pathway is considered to be the most crucial regulator. Accumulating evidence indicates that the p53 pathway participates in proliferation and apoptosis to regulate radiosensitivity in various cancers (Concin et al., 2000; Oei et al., 2015; Granados-López et al., 2021). In p53 dependent apoptosis, p53 promotes apoptosis by activating the transcription of apoptotic factors including Bax, PUMA, Noxa at the same time it can inhibit the transcription of anti-apoptotic factors such as Bcl-2, Bcl-xL, Survivin. On the other hand, p53 can accumulate in the cytoplasm and directly connect to the mitochondria, leading to changes in the permeability of the mitochondrial membrane.

By interacting with p53 pathway factors, LncRNAs regulate radiosensitivity. Under hypoxic conditions, p53 participates in the degradation of HIF-1α (Ravi et al., 2000). Fu et al. (2015) found that HIF-1α increased radiation resistance in cervical cancer cells by inhibiting the expression of p53 and increasing the expression of VEGF. Li N. et al. (2018) found that radiation reduced lncRNA levels and HIF-1α levels in mice bearing HeLa cells as well as in HeLa cells and C33A cells, and they carried out a series of experiments and finally revealed that overexpression of lncRNA HOTAIR induces radiation resistance by increasing the expression of HIF-1α in cervical cancer cells. The lncRNA DINO is a p53 transcriptional target and functional modulator (Sharma et al., 2020). LncRNA DINO can bind to TP53 to stabilize and upregulate transcriptional target genes of TP53. The lncRNA DINO has been found to be a potent tumor suppressor in specific subsets of human and mouse tissues (Marney et al., 2022). However, the relationship between DINO and radiation sensitivity has not been revealed. From previous research, we infer that DINO may have a positive effect on radiosensitivity.

LncRNA GAS5 is reported to connected with cancer cell apoptotic to affect radiosensitivity. Gao et al. (2019) collected twenty cervical cancer patient biopsy tumor samples before RT. These patients had never received any chemotherapy before radiation therapy. The twenty patients were divided into two groups: 1) radioresistant (9 cases) and 2) radiosensitive (11 cases). Researchers compared the expression of related molecules between the two groups. And found lncRNA GAS5 and miR-106b expression was significantly different between radioresistant tissues and radiosensitive tissues. Then, they used SiHa and ME180 cervical cancer cells to investigate the biological role of GAS5 in the radiosensitivity of cervical cancer. They found that upregulation of GAS5 enhanced radiosensitivity in SiHa cells, while downregulation of GAS5 decreased radiosensitivity in ME180 cells. GAS5 acted as a miR-106b sponge, inhibiting miR-106b and promote IER3 expression increasing cervical cancer cell radiosensitivity both *in vitro* and *in vivo*. IER3 is necessary for cervical cancer cell apoptotic activity by inhibiting the expression of BCL-2 and BCL-xL (Yoon et al., 2009). Fang et al. (2020) illustrated the relationship between GAS5 expression and the overall survival (OS) of cervical cancer patients using Kaplan–Meier analysis. They confirmed that poor OS was associated with low GAS5 expression. Univariate analysis indicated that GAS5 could be an independent prognostic factor for patients with CC. These above studies indicate that lncRNA GAS5 may become an important topic for cervical cancer clinical research, and researchers could detect these lncRNAs to infer the effect of RT in cervical cancer patients.

A majority of miRNAs play key roles in regulating radiation-induced apoptosis. In various cancers, lncRNAs function as miRNA sponges to regulate target gene expression. In recent studies, lncRNAs were reported to regulate radiosensitivity in cervical cancer cells by working as miRNA sponges. For example, in cervical cancer tissues and cells, the lncRNA SNHG6, which is a small nucleolar RNA host gene (SNHG family), was upregulated, knockdown of SNHG6 can improve the radiosensitivity of cervical cancer cells by promoting radiation-induced apoptosis. SNHG6 can directly sponge miR-485-3p.

Researchers have mentioned that in cervical cancer, miR-21 could facilitate cell proliferation by downregulating the expression of programmed cell death 4 (PDCD4) (Yao et al., 2009) as well as in breast cancer. MiR-21 inhibits the expression of p53-regulated genes. In various tumors, including non-small cell lung cancer (Song et al., 2017), and esophageal squamous cell carcinoma (ESCC) (Li F. et al., 2018; Lin et al., 2020), miR-21 has been shown to be related to radiation sensitivity. In cervical cancer, Zhang L et al. (2020) found that lncRNA MEG3 was negatively related to miR-21 to affect cell proliferation and apoptosis. These studies revealed that lncRNA MEG3 may also sensitize cervical cancer cells to radiation treatment by affecting the expression of miR-21, and this hypothesis needs to be proven. There are also many miRNAs that can interact with lncRNAs to affect the radiation effect in cervical cancer.

**FIGURE 3:**
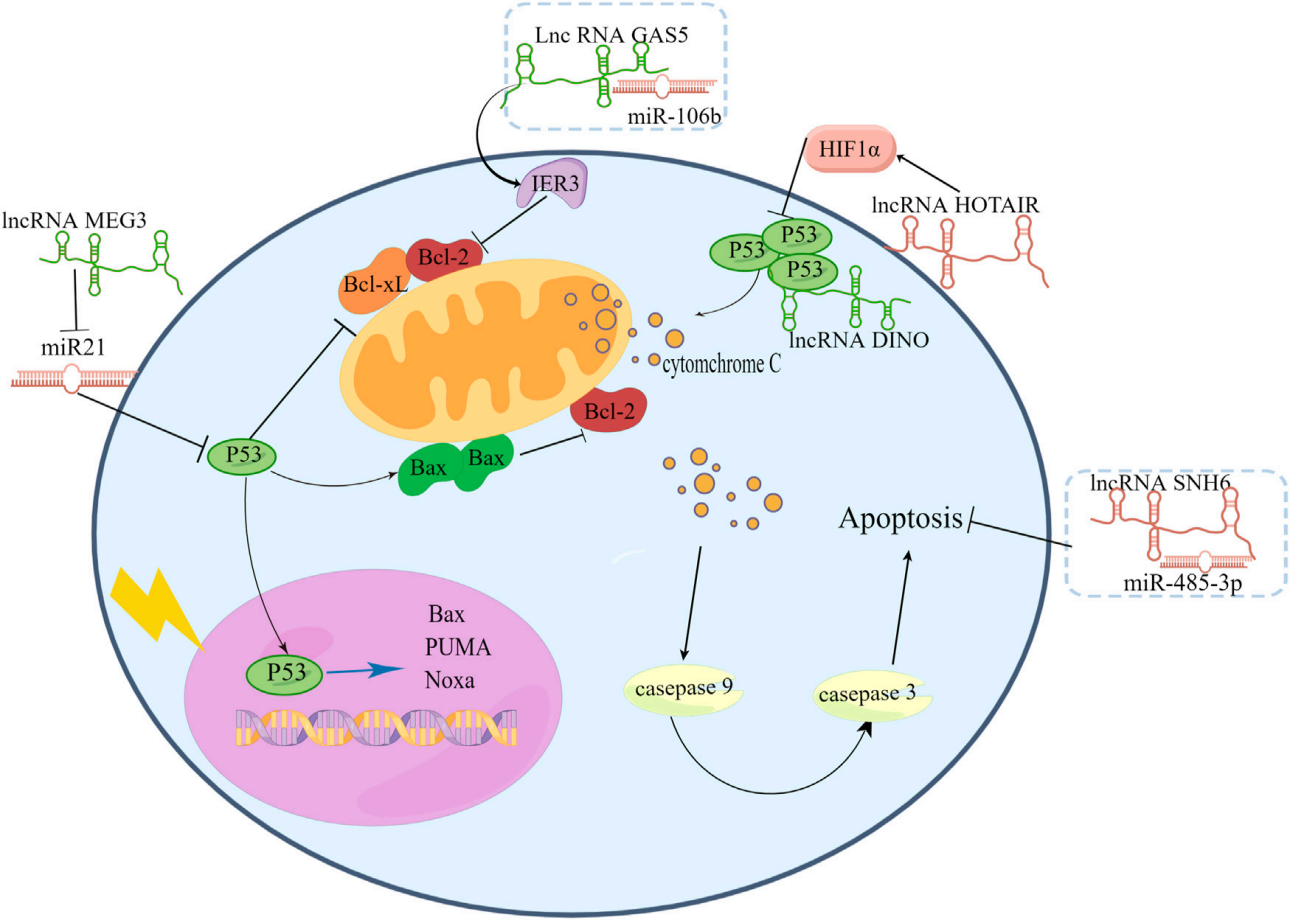
LncRNAs affect signaling pathways such as p53 or miRNA sponges to affect cervical cancer cell radiosensitivity.

Above all, lncRNAs are believed to affect cervical cancer cell radiosensitivity through their ability to induce apoptosis. Several mechanisms can be employed to achieve this effect, including affecting signaling pathways such as p53 or miRNA sponges that manipulate the expression of target genes (Figure 3). Hence, we can identify the difference between apoptotic and nonapoptotic cervical cancer cells by detecting noncoding RNAs in the future. Their relationship with RT sensitivity was studied, and a corresponding database was created for further exploration of the relationship between these noncoding RNAs and apoptotic signaling and to identify genes that can regulate the expression of these noncoding RNAs and therefore achieve RT sensitivity regulation.

## LncRNAs modulate EMT and CSCs

EMT plays an important role during tumor development. Tumor cells acquire an invasive phenotype when EMT programs aberrant activation. When tumor cells accept radiation, they may acquire stemness and oncogenic metabolism by inducing EMT (Lee et al., 2017). In tumors, there are cells that can self-renewing called CSCs. CSCs are similar to multipotent embryonic stem cells and possess self-renew capabilities (Reya et al., 2001). . Under specific conditions, CSCs and non-CSCs can convert to each other. CSCs play a crucial role in tumorigenesis and metastasis (Ponti et al., 2005). Researchers have shown that CSCs are generated by EMT (Mani et al., 2008). CSCs existence in cervical cancer with CD44+CK17+/sphere-forming and express stemness-related genes (Oct-4, Sox2 and so on) (Feng et al., 2009). In cervical cancer CSCs, EMT marker vimentin is high expression and perform a higher level of radioresistance than normal cervical cancers (López et al., 2012). And in HeLa, high expression of EMT marker Twist can induce EMT and elevate the expression of CD44 which is cancer stem marker of CSCs (Li and Zhou, 2011). These data indicate that there is a tight link between EMT and CSCs.

**FIGURE 4:**
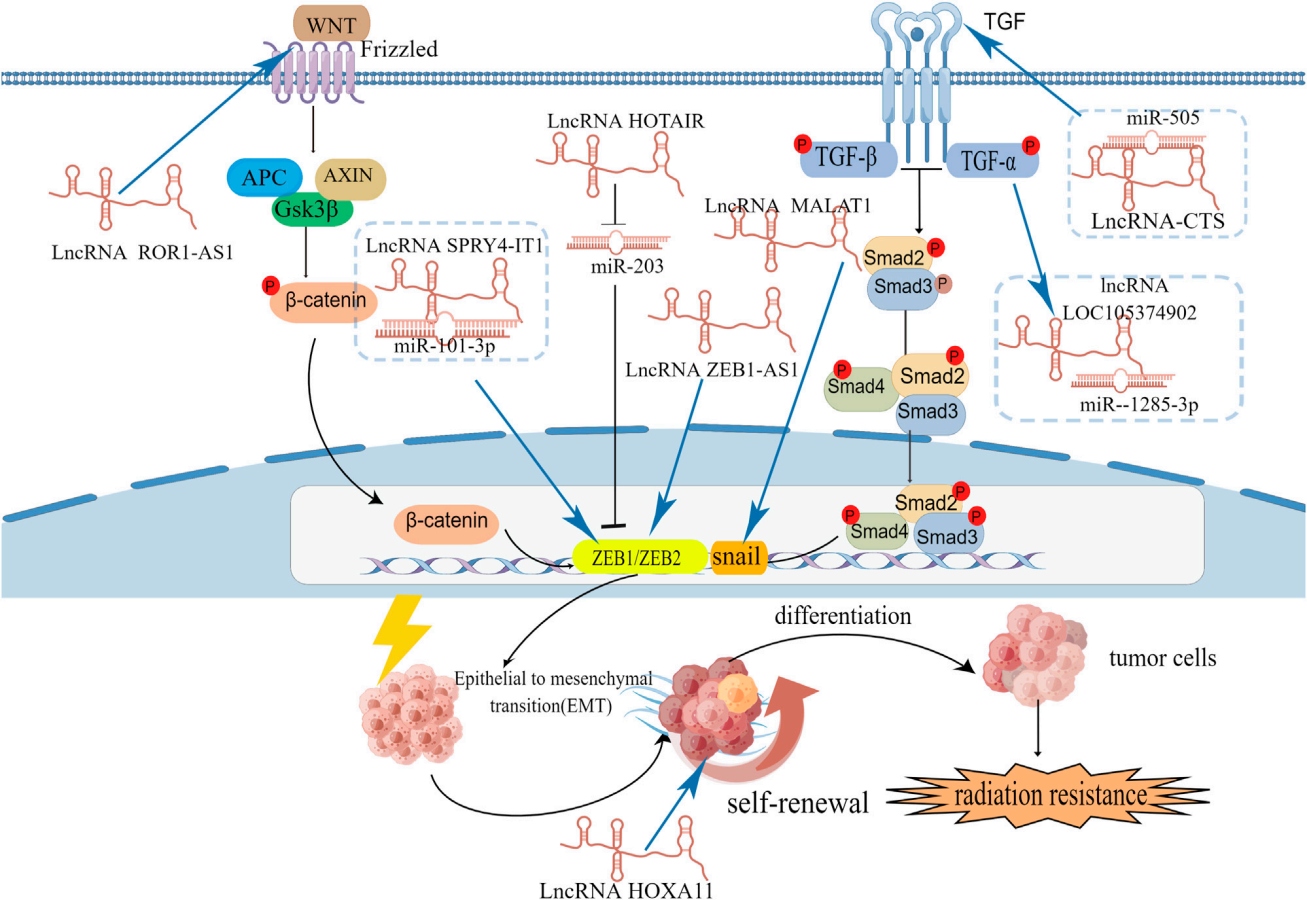
The signaling pathways and EMT-related transcription factors involved in lncRNAs and EMT.

In cervical cancer, lncRNAs can regulate EMT-related gene and transcription factors (Figure 4). For example, lncRNA NEAT1 can downregulate miR-361, which can inhibit EMT process in cervical cancer cells by targeting on EMT activator HSP90 (Xu et al., 2020). LncRNA ZEB1-AS1, SPRY4-IT1, MALAT1 and TUG1 are high-expression in cervical cancer cells and associated with poor prognosis. Overexpression of ZEB1-AS1 can increase the level of N-cadherin and vimentin and increase EMT promoter ZEB1 to facilitate EMT process (Cheng et al., 2018). Lnc RNA SPRY4-IT1 and HOTAIR can also target on ZEB1 to promote EMT process. SPRY4-IT1 can bind to miR-101-3p to down regulate its target gene ZEB1 (Fan et al., 2019). HOTAIR can inhibit the expression of miR-203 which is negatively correlated with ZEB1. LncRNA MALAT1 has a positive influence on EMT transcription factor snail and promote the EMT process (Sun et al., 2016). Knockdown the expression of lncRNA TUG1 can down regulate EMT-related markers (fibronectin, vimentin, and cytokeratin) (Hu et al., 2017).

LncRNAs are involved in the EMT process of cervical cancer cells by different signaling pathways (Figure 4). LncRNA tyrosine protein kinase transmembrane receptor one antisense RNA 1 (ROR1-AS1) is high-expression in cervical cancer cells and tissues. Downregulate ROR1-AS1 in cervical cancer can inhibit EMT-related markers N-cadherin and vimentin by influencing Wnt/β-catenin signaling pathway (Zhang J et al., 2020). LncRNA-CTS is high-expression in cervical cancer tissues and can bind to miR-505 to enhance EMT process by activating the TGF/SMAD pathway (Feng S. et al., 2019). TNF-α can upregulated lncRNA LOC105374902 by promoting it binding to STAT3. LncRNA LOC105374902 promote EMT process in cervical cancer by sponging miR-1285-3p (Feng Y. et al., 2019).

Increasing evidence indicates that lncRNAs may regulate CSCs in various cancers, including liver cancer (Wang et al., 2015; Ding et al., 2016), colorectal cancer (Tang D. et al., 2019), non-small cell lung cancer (Wu and Wang, 2017). In cervical cancer lncRNA HOXA11-AS has an association of EMT/CSCs. Compared with normal tissues, LncRNA HOXA11-AS is highly expressed in cervical cancer cells and has a negative effect on overall survival. Overexpression of LncRNA HOXA11-AS promotes sphere formation, CSC markers CD133+/CD44+ and self-renewal of cervical cancer cells. Knockdown of HOXA11-AS can downregulate the EMT-related genes (β-catenin, vimentin, snail) and stemness genes (SOX2, Oct-4, Nanog). At the same time, sphere-forming capacity of cervical cancer cells is also decreased. *In vivo*, knockdown of HOXA11-AS can decrease tumor growth. From this study, we predict that LncRNA HOXA11-AS plays an important role in the establishment of EMT/CSCs in cervical cancer cells (Kim et al., 2016). And in gastric cancer cells overexpression of LncRNA HOXA11 can also promote the expression of cancer stem markers including CD44, CD90, CD133, and Bmi1 as well as pluripotency markers Nanog and Sox2 (Wang C et al., 2019). These data indicate LncRNA HOXA11 may be a promising therapeutic target for the treatment of cancers.

According to the cancer stem cell theory, the resistance of cancer to chemotherapy and RT is due to resident CSCs (Wang et al., 2013). Radiation treatment can directly kill the majority of tumor cells by inducing apoptosis. However, a small portion of tumor cells can endure treatment, exhibit radioresistant properties and dedifferentiate. These tumor cells transform into CSCs *via* EMT (Chi et al., 2017). This shift leads to radioresistance in tumor cells, and CSCs can lead to cancer recurrence and metastasis. In cervical cancer, high levels of CSCs predict poor outcomes after radiotherapy (Fu et al., 2018). Blocking irradiation-induced CSCs activation may increase radiosensitivity in cervical cancer (Prabakaran et al., 2019). LncRNA HOTAIR has been proved to be necessary for EMT and stemness maintenance of cancer cell lines (Pádua Alves et al., 2013; Łuczak et al., 2016). In breast cancer, overexpression of HOTAIR can downregulate miR-34a expression and at the same time, can promote p53 binding to the p21 promoter and enhance CSC-MCF7(enrich the CSCs subpopulation from MCF7) proliferation, colony formation and migration. Overexpression of HOTAIR can upregulate Sox2, which is the key transcription factor regulate that regulates self-renewal capacity, and epigenetic expression of miR-34a can disturb this regulation (Deng et al., 2017). In cervical cancer, the lncRNA HOTAIR is also connected with stemness acquisition. Knockdown of the expression of HOTAIR can downregulate the expression of six stem cell markers, including NANOG, Oct4, CD44, ALDH1, CD133, and *vice versa* (Zhang et al., 2021). Compared with normal cervical epithelial cells (NCECs), the expression level of HOTAIR was significantly upregulated in cervical cancer cells such as HeLa and C33A cells (Li N. et al., 2018). High HOTAIR expression was correlated with shorter overall survival (Kim et al., 2015). The same outcome has been reported by other researchers. Initially, they used serum samples to detect the different levels of HOTAIR between cervical cancer patients and normal women. They found that circulating HOTAIR levels were significantly higher in cervical cancer patients and connected with a poor prognosis. Then, they performed further research to investigate the biological functions of HOTAIR in cervical cancer. They used four cervical cancer cell lines to detect HOTAIR expression and carried out a series of experiments, such as MTT assays and migration and invasion assays. They finally concluded that HOTAIR acts as an oncogene in cervical cancer and then cultured primary cervical cancer cells from 25 fresh cervical cancer tissues to investigate the relationship between HOTAIR and the response to RT. They demonstrated that the expression level of HOTAIR was negatively correlated with that of P21. By hindering p21 expression in HeLa cells, HOTAIR can induce radioresistance (Jing et al., 2015). This result is consistent with the results of the above breast cancer studies, HOTAIR is also reported to be connected with radiosensitivity in colorectal cancer (Yang et al., 2016; Liu Y. et al., 2020), pancreatic cancer (Wu et al., 2018), and liver cancer (Zhai et al., 2020). Therefore, HOTAIR may be a potential target against cervical cancer.

However, very few studies have investigated the link between lncRNAs and CSCs in cancer RT, and more *in vitro* experimental data are needed. With suitable experimental methods, *in vivo* experimental data would be more convincing. It may be possible to develop new methods to improve therapeutic effects of radiotherapy in cervical cancer by gaining a deeper understanding of lncRNAs, CSCs and EMT.

## LncRNAs regulate aerobic glycolysis

Malignant tumors have a high aerobic glycolysis rate, leading to high lactic acid production, and this phenomenon is called the Warburg effect. Aerobic glycolysis is necessary for cancer cell proliferation (Vander Heiden et al., 2009). Lactate is the product of aerobic glycolysis and is necessary for cancer cell migration and metastasis (San-Millán and Brooks, 2017). In previous studies, it has been found that glycolysis increases radiosensitivity (Quennet et al., 2006; Shimura et al., 2014). The enzyme pyruvate kinase isozyme type M2 (PKM2), which produces ATP, is a rate-limiting enzyme at the end of glycolysis (Wang H et al., 2019). To investigate the role of PKM2 in the RT of cervical cancer, Lin et al. (2019) collected human tissue samples from 94 patients who accepted radiation therapy. According to the response after the completion of RT, 94 patients were divided into two groups: a complete response (CR) and a noncomplete response (nCR) group. They carried out IHC staining to detect the expression of PKM2 in these two groups and found that the expression of PKM2 in the nCR group was higher than that in the CR group, and PKM2 expression was enhanced in cervical cancer cells after radiation. Then, they used HeLa and SiHa cell lines with stable low PKM2 expression to demonstrate that knocking down PKM2 can enhance the radiosensitivity of cervical cancer cells. Luan and Wang (2018) found that in cervical cancer cells, SiHa and Caski, lncRNA XLOC_006390 were related to PKM2. Puckett et al. (2021) summarized the relationship between noncoding RNAs and PKM2. All these studies revealed that lncRNAs may affect the efficiency of RT by influencing the expression of PKM2.

Like PKM2, hexokinase 2 (HK2) is another rate-limiting enzyme during aerobic glycolysis. LncRNA urothelial cancer associated 1 (UCA1) was reported to be connected with HK2 in cervical cancer. LncRNA UCA1 first discovered and researched in bladder cancer as an oncogene (Wang et al., 2008). Fan et al. (2018) explored the role of the lncRNA UCA1 in cervical cancer radioresistance. They establish irradiation-resistant (IRR) cervical cancer cell lines, SiHa–IRR and HeLa–IRR. The expression of UCA1 was significantly increased in SiHa–IRR and HeLa–IRR compared with SiHa and HeLa. To explore the function of lncRNA UCA1 in regulating the therapeutic effects of radiotherapy in cervical cancer, they carried out siRNA knockdown and plasmid overexpression assays. And they revealed overexpression of LncRNA UCA1 lead to radioresistance in cervical cancer. And in SiHa–IRR and HeLa–IRR glycolysis related proteins (HK2 and PKM, HIF-1α) were increased. Then the glycolysis inhibitor 2-dG which compete with glucose for HK2 is used in SiHa–IRR and HeLa–RR to detect the radiosensitivity. 2-dG improves the radiosensitivity of SiHa–IRR and HeLa–IRR. These findings suggest that LncRNA UCA1 affect the effects of radiotherapy by the HK2/glycolytic pathway in cervical cancer cells. The abnormal activation of glycolysis may have a role in cervical cancer radioresistance. Inhibition of glycolysis restored the radiosensitivity of irradiation-resistant cervical cancer cells. Although further research regarding the mechanism underlying the regulatory effects of lncRNAs regulating aerobic glycolysis to affect radiation is needed, existing studies have expanded the understanding of the role of aerobic glycolysis in radioresistance and provided potential novel targets to enhance radiosensitivity in cervical cancer. Targeting glycolysis may provide an improved radiotherapy for cervical cancer.

## Other factors regulating RT sensitivity

Human telomerase reverse transcriptase (hTERT) is a subunit of telomerase capable of preserving telomere integrity. Cancer cells contain a highly active promoter of hTERT, whereas most normal cells contain a weak promoter (Shay and Wright, 2011). Barczak et al. (2018) noted that in head and neck cancer cell lines, inhibiting hTERT expression can induce apoptosis and result in G1/S/G2 cell cycle arrest, thus enhancing the response to RT. This study reminds researchers that hTERT may be an important target to improve the efficacy of RT in malignancy. In 2020, Li et al. (2020) reported that in cervical cancer, binding of HMGB3 to the hTERT promoter promotes the transcriptional upregulation of hTERT and promotes cell growth and radioresistance. HMGB3 is the most abundant nonhistone protein in eukaryotes (Nemeth et al., 2006). Researchers have used bioinformatics and experimental analyses of several cell lines and identified an antisense transcript upstream in the human telomerase reverse transcriptase (hTERT) promoter region named hTERT antisense promoter-associated (hTAPAS) RNA. By analyzing The Cancer Genome Atlas (TCGA), they found that the expression of hTERT was negatively correlated with that of hTAPAS (Malhotra et al., 2018). Hu et al. (2015) reported that in esophageal squamous cell carcinoma, lncRNA CDKN2B-AS1 has a positive correlation with hTERT. Therefore, we suggest that in tumor cells, special lncRNAs may affect the efficacy of RT by regulating hTERT.

## Conclusion and future perspectives

Radiotherapy is a key treatment modality for cervical cancer, but the efficacy is limited by radioresistance. Emerging research has demonstrated that lncRNAs are associated with radioresistance, and the relationship between them has gradually become a research hotspot. Studies have shown that lncRNAs have potential for use as biomarkers of the response to RT and can modulate the RT response of cervical cancer.

The radiation response of cervical cancer differs greatly among different patients; thus, it is important to predict the response to radiation treatment for each patient. In addition, the overall RT treatment time for cervical cancer patients is usually several weeks. Thus, early identification of patients who are not responding to therapy would provide an opportunity to consider alternative treatment options. This would benefit individualized treatment. However, it is difficult to distinguish different lncRNAs that correlate with only the RT response between responders and nonresponders because in clinical studies, patients usually undergo combined chemoradiotherapy. Fortunately, a limited number of recent cervical cancer studies have shown that lncRNAs indeed exhibit differences in the setting of RT alone. At present, there are few studies on the ability to predict the RT effect of cervical cancer based on lncRNAs, but this will be a hot topic that requires large-scale clinical research.

Studies have focused on the effect of the expression level of lncRNAs on the radiosensitivity of cervical cancer *via* basic experiments. However, the molecular mechanisms underlying radioresistance have not yet been completely elucidated. In this review, we summarized the potential mechanisms of lncRNAs that are associated with radiosensitivity in cervical cancer (Table 1). The role of different lncRNAs in the RT sensitivity of different malignant tumors could be twofold. Some can enhance radiosensitivity, while others may enhance radioresistance. Therefore, it is of great significance to identify abnormally expressed lncRNAs in cervical cancer and to investigate their function and potential related signaling pathways in the response to RT. In this way, researchers can develop more effective radiotherapies and institute individualized treatment.

**TABLE 1:** LncRNAs and radiotherapy in cervical cancer.

LncRNA	Expression in cervical cancer	Radiosensitivity	Mechanism	References
NEAT1	Increased	Decreased	NEAT1 competitively binds miR-193b-3p to upregulate the expression of cyclin D1	Han et al. (2018)
LINC00662	Increased	Decreased	LINC00662 adsorpt miR-497-5p and upregulation of CDC25A	Wei et al. (2020)
LINP1	Increased	Decreased	LINP1 knockdown enhanced cell apoptosis and delayed repairs of DNA DSBs after radiation	Wang et al. (2018)
MALAT1	—	Decreased	Sponges miR-145 and enhance G2/M phase block	Lu et al. (2016)
GAS5	Decreased	Increased	miR-106b/IER3	Gao et al. (2019)
SNHG6	Increased	Decreased	LncRNA SNHG6 sponges miR-485-3p to release STYX.	Liu et al. (2020a)
HOTAIR	Increased	Decreased	HOTAIR inhibiting p21 in HeLa cells	Jing et al. (2015)
UCA1	—	Decreased	UCA1 regulates radioresistance through the glycolytic pathway by modulating HK2 in cervical cancer	Fan et al. (2018)
PCAT1	Increased	Decreased	Sponges miR-128	Ge et al. (2020)

The study of lncRNAs in RT in cervical cancer remains in the initial stage, and most existing studies have only been carried out *in vitro*; thus, clinical trials are lacking. Moreover, the particular molecular biological mechanisms by which lncRNAs regulate radiosensitivity have not yet been completely described.

Understanding the molecular pathways regulated by lncRNAs should greatly improve the effectiveness of RT in cervical cancer and reveal better treatment strategies. Through summarizing the existing research articles, it was found that lncRNAs mainly affect RT sensitivity by affecting the DNA damage repair, cell cycle, apoptosis, EMT, CSCs, and glycolysis in cervical cancer RT. Recent studies have reported that human telomerase reverse transcriptase may also be related to the effect of RT, which is a very important finding and needs to be verified by more experiments. After recognizing the role of lncRNAs in RT of cervical cancer, we need to conduct *in vivo* experiments to verify these conjectures. We believe that research on lncRNAs in the response to RT will provide new RT sensitization strategies for cervical cancer. Studies on lncRNAs that affect the cervical cancer RT response will provide new sensitization strategies for cervical cancer RT and lay the foundation for the clinical application of lncRNAs in the future.

## Abbreviations

RT, radiotherapy; lncRNAs, long noncoding RNAs; CSCs, cancer stem cells; EMT, epithelial–mesenchymal transition; ATM, ataxia telangiectasia-mutated; ATR, ATM and Rad-3 related; NHEJ, nonhomologous end joining; PKM2, pyruvate kinase isozyme type M2; HK2, hexokinase 2; hTERT human telomerase reverse transcriptase.
